# Case report: Successful treatment of anti‐MDA5‐positive to negative dermatomyositis‐associated interstitial lung disease with the JAK inhibitor tofacitinib

**DOI:** 10.1002/iid3.897

**Published:** 2023-06-14

**Authors:** Zong Jiang, Xiaoling Yao, Fang Tang, Wukai Ma

**Affiliations:** ^1^ Second Clinical Medical College Guizhou University of Traditional Chinese Medicine guiyang China; ^2^ Department of Internal Medicine The Second Affiliated Hospital of Guizhou University of Traditional Chinese Medicine Guiyang China

**Keywords:** Anti‐MDA5 antibody, dermatomyositis, interstitial lung disease, JAK inhibitor, tofacitinib

## Abstract

**Objective:**

Anti‐MDA5 antibody‐positive dermatomyositis (DM) is a rare clinical autoimmune disease, and anti‐MDA5‐positive DM with interstitial lung disease (ILD) is the most important cause of death in DM patients. We reported the efficacy of the JAK1/3 inhibitor tofacitinib as an anti‐MDA5‐negative treatment option for patients with anti‐MDA5‐positive DM‐ILD.

**Method and process:**

Here we report a 51‐year‐old female patient with cough, sputum, shortness of breath for 5 months, rash for 3 months, and muscle pain in the extremities for 1 month. After conventional immunosuppressive therapy plus hormone therapy, the remission was slow. Methylprednisolone was successfully reduced after we administered tofacitinib and tacrolimus. After 132 weeks of follow‐up, anti‐MDA5 antibody turned negative, clinical symptoms were relieved, and lung Imaging tests were successfully reversed.

**Results and Conclusion:**

There is currently no report of tofacitinib supplement therapy for anti‐MDA5 positive to negative DM. With this case report, tofacitinib is an option for the treatment of anti‐MDA5‐positive DM‐ILD, which deserves attention.

## Graphical abstract

We have observed for the first time that tofacitinib is used in the treatment of demetomyositis with negative anti MDA5 antibodies.

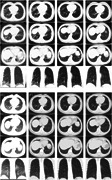

## 1 INTRODUCTION

Dermatomyositis (DM) is an autoimmune disease with clinical manifestations of muscle and skin damage, often accompanied by multi‐system involvement, especially in the lungs.^1^ Positive anti‐MDA5 antibodies are highly associated with interstitial lung disease (ILD), which is one of the major mortality factors in DM patients.^2^ Current conventional drug treatment for DM‐ILD includes glucocorticoids, immunosuppressants, and anti‐pulmonary fibrosis drugs.^3^ Long‐term use of glucocorticoids can lead to many adverse reactions, a high risk of immunosuppressive infections, unpredictable adverse drug reactions, and the duration of treatment with anti‐pulmonary fibrosis drugs is not easy to manage, resulting in DM‐ILD patients, especially those with positive anti‐MDA5 antibodies. The effective treatment rate is low. As a small‐molecule targeted drug, many studies have confirmed the effectiveness of tofacitinib in the treatment of patients with anti‐MDA5‐positive DM‐ILD.^4^, ^5^ However, there is no report of the negative conversion of anti‐MDA5 antibodies by tofacitinib treatment. Here we reported a case of anti‐MDA5 antibody‐positive DM‐ILD patients who were treated with hormones and immunosuppressive agents in combination with tofacitinib and had negative anti‐MDA5 antibodies, which is summarized below for clinical reference.

## 2 CASE REPORT

**Figure 1:**
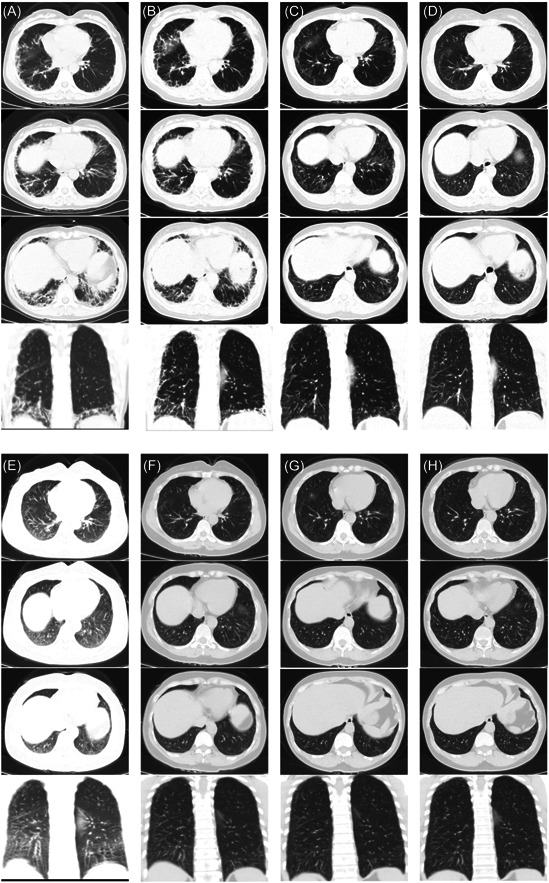
From (A to H), changes in lung imaging at 0, 12, 36, 64, 72, 84, 108, and 132 weeks of treatment, respectively, and the film‐glass density shadow, patch shadow and grid shadow continued to disappear.

A 51‐year‐old female patient with a 10‐year history of psoriatic arthritis developed a paroxysmal cough and cough with white, sticky sputum after a cold 5 months ago, accompanied by chills, general malaise, chest tightness, shortness of breath, back pain and hoarseness after exercise. She did not get any better after considering cold treatment. 2 months later, feeling shortness of breath after activity and speech was worse than before, and there was scattered erythema on the neck, some of which merged into a piece, rough skin on the radial side of the right index finger, the distal end of the hands, and white skin after cold, a chest CT was performed at an external hospital. Interstitial infection in the right lobe, the lingual segment of the upper lobe of the left lung, and the lower lobes of both lungs were seen, and electronic bronchoscopy showed no abnormalities. Pulmonary function tests showed moderate restrictive ventilatory dysfunction, small airway dysfunction, and moderate diffusion impairment. The electromyography showed a trend of myogenic injury in the proximal muscles of the extremities. Bilateral interstitial pneumonia and connective tissue disease were considered. To combat the infection, methylprednisolone 40 mg once daily and pirfenidone 200 mg three times daily was used for treatment, and the cough and sputum improved, with no improvement in shortness of breath after exercise, without further deterioration. After 4 months of activity, chest tightness, shortness of breath, cough and white sputum appeared. She went to the Department of Respiratory Medicine, West China Hospital, Sichuan University for examination of anti‐Mi‐2α antibody, anti‐MDA5 antibody and anti‐PM Scl antibody (+−). CT scan of the chest revealed vitreous opacities, patchy shadows, and grid‐like shadows in both lung membranes, mostly due to interstitial inflammation in both lungs. Biopsy of the left deltoid muscle DM skeletal myopathy could not be excluded, followed by methylprednisolone modulation of immunity and no significant relief of symptoms after anti‐infection. We considered our department to check the positive anti‐MDA5 antibody to diagnose DM, and to check the finger pulse. Oxygen 89% (no oxygen inhalation), dry and wet rales could be heard, a small amount scattered in the lower lungs, veclro rales could be heard in the bases of both lungs, chest CT showed chronic interstitial changes in both lungs; mixed type in the lower lobes of both lungs (Figure 1). Possibility of infection, fungus, procalcitonin, C‐reactive protein, erythrocyte sedimentation rate, muscle enzyme spectrum, electrolytes, thyroid function, tumor markers were normal, excluding other autoimmune diseases and tumors, sputum culture was Lewy persistent bacillus infection, antibiotic anti‐infective treatment and methylprednisolone 40 mg qd (reduced by 4 mg per week), cyclosporine 25 mg bid, pirfenidone 200 mg tid treatment, cough, sputum, chest tightness, shortness of breath, muscle pain significantly improved, and no skin damage.

After 12 weeks of treatment, the patient visited Peking Union Medical College Hospital and was found to be anti‐MDA5 positive. She was treated with methylprednisolone 12 mg qd, tacrolimus 2 mg qd, compound cyclophosphamide 50 mg qd, and pirfenidone 200 mg tid. No rash was found after treatment. Muscle pain was present, but chest tightness, shortness of breath, cough and sputum were felt.

**Figure 2:**
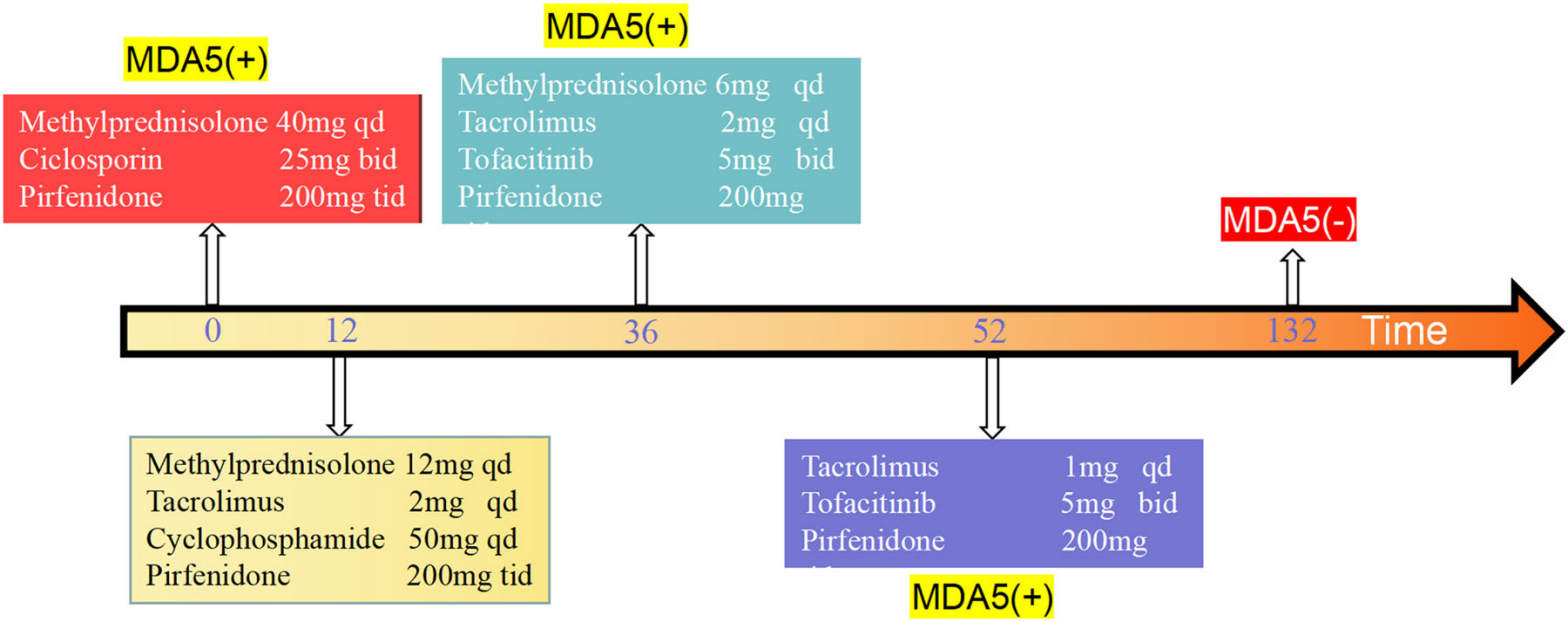
Timeline diagram of treatment regimen and follow‐up drug adjustments. qd: every day, bid: twice day, tid: three times a day.

**Figure 3:**
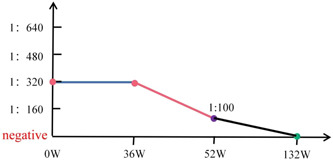
BCA method for detecting changes in anti MDA5 antibodies.

At week 36 of treatment, Considering patient's economic ability and the toxic side effects of cyclophosphamide, after excluding infection, tumor, tuberculosis, and hepatitis, we adjusted the treatment plan with methylprednisolone 6 mg qd, tacrolimus 2 mg qd, tofacitinib 5 mg bid, pirfenidone 200 mg tid. Chest tightness and shortness of breath, cough, and expectoration continued to relieve, and hormones were successfully reduced at 52 weeks of treatment. Tacrolimus Capsules 1 mg qd remained in remission and dring the multiple follow‐up, the anti‐MDA5 antibody was positive for many times using the Cytometric Bead Array detection method, but the titer gradually decreases (Figure 2 and 3).

**Figure 4:**
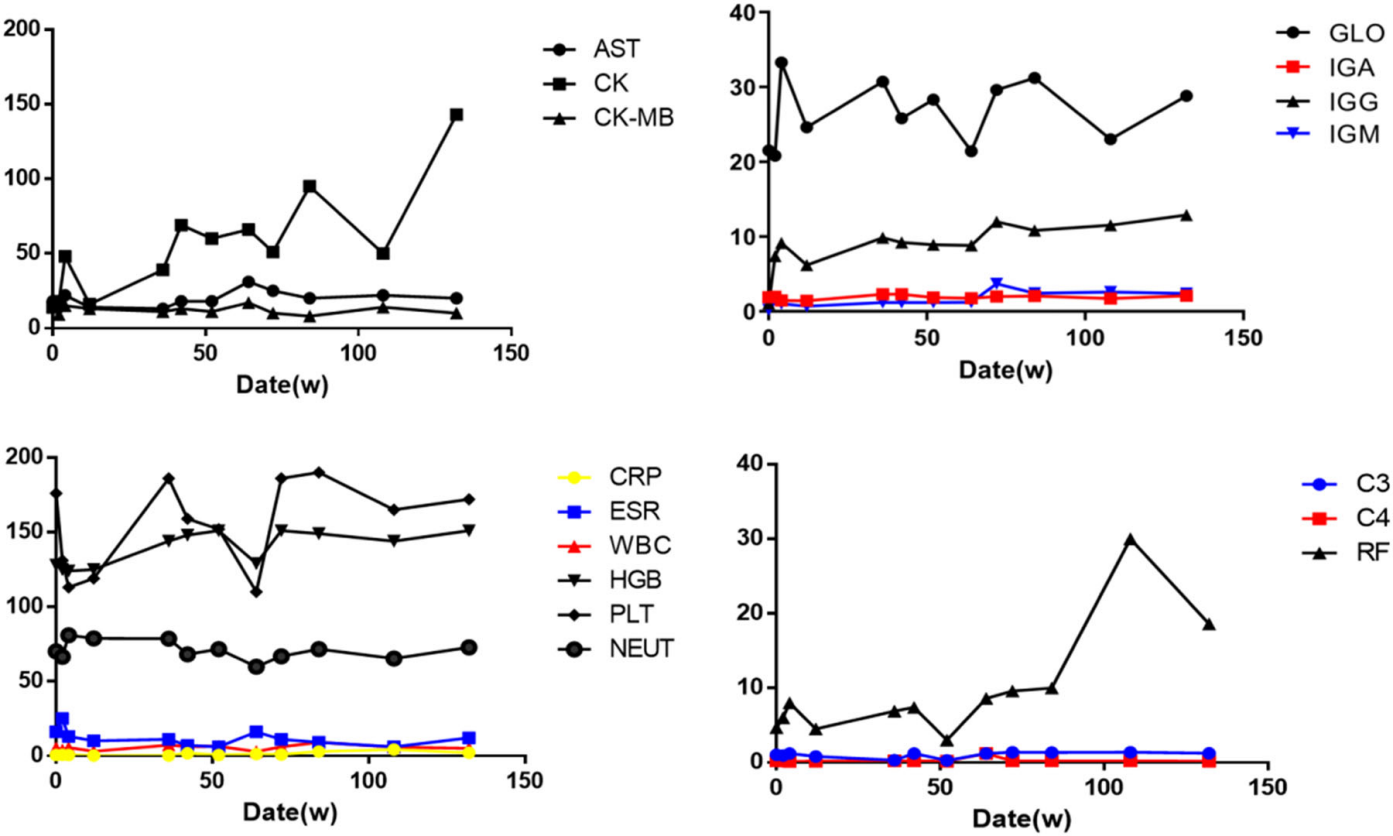
Clinical course and laboratory examination. AST, Aspartate aminotransferase; C3/4, complement 3/4; CK, Creatine Kinase; CK‐MB, Creatine kinase isoenzymes; CRP, C‐reactive protein; ESR, erythrocyte sedimentation rate; GLO, Globulin; IG, Immunoglobulins; HGB, hemoglobin; NEUT, neutrophil; PLT, platelet; RF, rheumatoid factor; WBC, white blood cell.

At the 132 weeks of follow‐up, we comprehensively evaluated the patient's condition again. Pulse oxygen was 95% (without oxygenation), and the pulmonary function was normal. After repeated checks, the anti‐MDA5 antibody turned negative (Figure 3), and the symptoms continued to relieve. First, we continued to administer tacrolimus Division 1 mg qd, tofacitinib 5 mg bid, pirfenidone 200 mg qd maintenance therapy (Figure 2). It was worth noting that the patient's muscle enzyme, immunoglobulin, C‐reactive protein, and erythrocyte sedimentation rate were normal from the onset to the 132‐week follow‐up (Figure 4).

## 3 DISCUSSION

Anti‐MDA5 antibody‐positive DM has a rapid onset, more severe disease and worse prognosis, and ILD is the leading cause of death in anti‐MDA5 antibody‐positive DM patients.^6^, ^7^ DM is a rare disease, and many drugs have been recommended for the treatment of DM, including glucocorticoids, methotrexate, tacrolimus, cyclosporine and intravenous immune globulin, which have been proven to be clinically effective.^8^ But when the above‐mentioned adverse reactions occur or are all ineffective, there are few drugs to choose from. Tofacitinib has been shown in many studies to have good efficacy in DM patients.^9^, ^10^ On this basis, we selected tofacitinib under the full informed consent of the patients and achieved good curative effect.

Reviewing this patient, although serum muscle enzymes were not elevated throughout, our diagnosis was considered despite the consistent absence of elevated serum myosin and the presence of fatigue, neck rash, electromyography suggestive of myogenic lesions, muscle biopsy suggesting inflammatory myopathy, changes in ILD, and positive anti‐MDA5 antibody. Anti‐MDA5 antibody was positive for DM. Initially, the patient responded well to treatment, but as the steroids were tapered, the patient's symptoms and imaging studies did not resolve significantly. Even with the addition of cyclophosphamide and tacrolimus, the patient did not experience significant relief. But we attempted tofacitinib because of tacrolimus' prolonged usage and the negative effects of steroid hormones, and we were able to successfully reduce and cease using steroid hormones after taking tofacitinib for 16 weeks. The anti‐MDA5 antibody was effectively turned negative after 96 weeks, but the patient's symptoms persisted. Remission, lung CT and ILD manifestations were successfully reversed. It was reported that patients with anti‐MDA5 antibody positive DM‐ILD were treated with rituximab, tofacitinib and pirfenidone,^11^ and the anti‐MDA5 antibody was transformed after 76 weeks of treatment. However, since tofacitinib was used after rituximab treatment, it could not be well explained which drug was anti‐MDA5 antibody transformation, but tofacitinib played a very good role in maintenance treatment. Therefore, we believed that tofacitinib has a potential role in the maintenance therapy of anti‐MDA5 antibody‐positive DM‐ILD.

In conclusion, we reported a patient with anti‐MDA5 antibody‐positive DM‐ILD and concluded for the first time that tofacitinib may be an option for anti‐MDA5 antibody‐positive conversion during maintenance therapy. However, more clinical and experimental studies are needed for further confirmation.

## AUTHOR CONTRIBUTIONS

Zong Jiang actively wrote the manuscript and created the fifigures. Fang Tang provided constructive advice and critically revised the manuscript. Xiaoling Yao edited the manuscript. Wukai Ma guided the treatment of the patient.

## CONFLICT OF INTEREST STATEMENT

The authors declare no conflict of interest.

## ETHICS STATEMENT

Informed patient consent was obtained for the publication of any potentially identifiable images or data contained herein. Throughout the clinical course, patients were fully informed about the treatment and potential side effects and agreed to the treatment.

## Data Availability

The original contributions presented in the study are included in the article/Supporting Information Material. Further inquiries can be directed to the corresponding authors.
